# Poster Session I - A31 ORAL MICROBIAL DEGRADATION OF FOOD ALLERGENS: IMPLICATIONS FOR PEANUT AND COW’S MILK ALLERGY

**DOI:** 10.1093/jcag/gwaf042.031

**Published:** 2026-02-13

**Authors:** P A Hall, B Barbosa da Luz, L Rondeau, R Dang, R Jiménez-Saiz, A Caminero

**Affiliations:** McMaster University, Hamilton, ON, Canada; McMaster University, Hamilton, ON, Canada; McMaster University, Hamilton, ON, Canada; McMaster University, Hamilton, ON, Canada; Universidad Autónoma de Madrid, Madrid, Spain; McMaster University Faculty of Health Sciences, Hamilton, ON, Canada

## Abstract

**Background:**

Food allergies affect 5-10% of Canadians, often leading to severe immune reactions and anaphylaxis to specific food proteins. Despite their prevalence, research on preventive treatments remains limited, and life-threatening anaphylaxis from accidental exposure remains a significant concern. Common allergens include peanut (PN) and cow’s milk (dairy) proteins. PN allergy is typically characterized by Th2-skewed, IgE-mediated immune responses to major allergens such as Ara h 1 and Ara h 2, while cow’s milk allergy often involves IgE reactivity to Bos d 5 and Bos d 11. These allergens are highly resistant to human enzyme degradation, contributing to their immunogenic properties. However, the orogastrointestinal microbiota, comprising trillions of bacteria, can degrade proteins that human enzymes cannot, suggesting that bacterial digestion may aid in allergen elimination and attenuation of IgE-mediated immune responses.

**Aims:**

Aim 1: To identify bacteria capable of breaking down PN and dairy allergens.

Aim 2: To test PN and dairy immunogenicity after bacterial digestion.

**Methods:**

Saliva samples collected from healthy volunteers were incubated on different agar media supplemented with PN or dairy proteins. Bacteria with the capacity to degrade these proteins macroscopically were selected and further tested to degrade immunodominant allergens Ara h 1, Ara h 2, Bos d 5 and Bos d 11 by ELISA (Inbio) and SDS-PAGE. Additionally, a collection of *Lactobacillus* was screened for their capacity to degrade the same allergens. Bacteria with the capacity to degrade PN were tested in a new mouse model of PN sensitization and challenge. Briefly, mice were sensitized intraperitoneally to PN and then challenged to PN previously digested with the bacteria of interest, or native PN.

**Results:**

A total of 81 bacteria belonging to *Staphylococcus*, *Rothia*, *Neisseria* and other minor genera were isolated from human saliva with the capacity to degrade PN/dairy allergens. *Lactobacillus* also contributed to peanut and dairy allergen degradation. *Rothia*, *Lactobacillus*, *Clostridium* and specific species of *Staphylococcus* degrade the immunodominant allergens Ara h 1 and 2, and Bos d 5 and Bos d 11. Furthermore, PN degradation by *Rothia* and *Clostridium* strains reduced mast cell degranulation compared to intact PN in a preclinical mouse model of IgE-mediated food allergy.

**Conclusions:**

Oral bacteria efficiently degrade food allergens thereby affecting their immunogenicity. This microbial activity may help explain the variability in peanut thresholds observed among allergic patients. Bacteria such as *Lactobacillus* and *Rothia* show potential for increasing allergen threshold or mitigating allergen cross-contamination in the food industry.

**Funding Agencies:**

NRCCanadian Allergy, Asthma, and Immunology Foundation (CAAIF)

